# A new perspective when examining maize fertilizer nitrogen use efficiency, incrementally

**DOI:** 10.1371/journal.pone.0267215

**Published:** 2022-05-11

**Authors:** Newell R. Kitchen, Curtis J. Ransom, James S. Schepers, Jerry L. Hatfield, Raymond Massey, Scott T. Drummond

**Affiliations:** 1 USDA-ARS Cropping Systems and Water Quality Research Unit, USDA/ARS, Columbia, Missouri, United States of America; 2 Department of Agronomy and Horticulture, University of Nebraska-Lincoln, Lincoln, Nebraska, United States of America; 3 USDA-ARS (retired), National Laboratory for Agriculture and the Environment, Ames, Iowa, United States of America; 4 Department of Agricultural and Applied Economics, University of Missouri, Columbia, Missouri, United States of America; 5 Corteva Agrisciences, Johnston, Iowa, United States of America; Ghazi University, PAKISTAN

## Abstract

For maize (*Zea mays* L.), nitrogen (N) fertilizer use is often summarized from field to global scales using average N use efficiency (NUE). But expressing NUE as averages is misleading because grain increase to added N diminishes near optimal yield. Thus, environmental risks increase as economic benefits decrease. Here, we use empirical datasets obtained in North America of maize grain yield response to N fertilizer (n = 189) to create and interpret *incremental* NUE (iNUE), or the change in NUE with change in N fertilization. We show for those last units of N applied to reach economic optimal N rate (EONR) iNUE for N removed with the grain is only about 6%. Conversely stated, for those last units of N applied over 90% is either lost to the environment during the growing season, remains as inorganic soil N that too may be lost after the growing season, or has been captured within maize stover and roots or soil organic matter pools. Results also showed iNUE decrease averaged 0.63% for medium-textured soils and 0.37% for fine-textured soils, attributable to fine-textured soils being more predisposed to denitrification and/or lower mineralization. Further analysis demonstrated the critical nature growing season water amount and distribution has on iNUE. Conditions with too much rainfall and/or uneven rainfall produced low iNUE. Producers realize this from experience, and it is uncertain weather that largely drives insurance fertilizer additions. Nitrogen fertilization creating low iNUE is environmentally problematic. Our results show that with modest sub-EONR fertilization and minor forgone profit, average NUE improvements of ~10% can be realized. Further, examining iNUE creates unique perspective and ideas for how to improve N fertilizer management tools, educational programs, and public policies and regulations.

## Introduction

Nitrogen is the most abundant element in the earth’s atmosphere and the nutrient used in the largest amount by plants. For grain crop production, efficiently using plant-available N from any source (fertilizer, manure, irrigation water, soil mineralization) is a challenge because nitrate-N is soluble in water and thus subject to losses via agricultural field runoff and leaching through porous soils. In addition, N can be lost to the atmosphere from waterlogged soils via denitrification. Excess water is the critical component in these N-loss processes, which implicates weather patterns and irrigation practices. Quantifying agricultural N losses has been the focus of numerous scientific investigations since the 1960s when scientists recognized that overapplication of inexpensive N fertilizer would eventually become an environmental problem, and it has [1–5]. Low fertilizer NUE is at the heart of this environmental concern [2, 3, 6–8] and it remains a challenge six decades later. The question at hand is what crops and practices account for the lowest NUE values and therefore create the greatest risk for the environment.

Maize accounts for more than 40% of the global production of the six leading grain crops (S1 Table). The global maize harvest contains over 16 million MT of N, which accounts for ~37% of the N used by the six leading grain crops. Maize N budgets show global fertilizer NUE ranges between 25 and 40% [7, 9–11], which implies that N fertilizer application rates require two to four times more N than contained in the harvested grain. In contrast, field research trials report fertilizer NUE of maize grain ranging from 10 to 60% [11–13]. When contrasted with other grains, maize NUE is often reported lower. Nitrogen fertilizer rates applied to maize are typically higher than for other grains because maize yields are typically higher. Further, no autoregulation exists to deter overapplications of N in maize since excess fertilization has little or no negative impact on crop performance, as can occur with other crops (e.g., lodging in wheat (*Triticum aestivum* L.)). The only major disincentive to maize producers for over applying is economical as related to N fertilizer costs [11]. In summary, the likelihood of N over application, and related economic and environmental implications, is most notable in maize compared to other crops.

Several approaches to quantify maize fertilizer NUE have been used over past decades (S2 and S3 Tables). Traditional fertilizer NUE calculations use yield and N fertilizer rate data for a given growing season. For maize, grain yield response to N most commonly follows a quadratic relationship, and if several N rates are included that are > the optimal N rate, a response will likely follow a quadratic-plateau trend [14]. Yield at each N rate can be used to calculate NUE (e.g., S3 Table), but typically NUE values are reported based on the maximum application rate. Expressed this way, it represents an average NUE. However, as yield response exhibits diminishing marginal returns to increasing N fertilizer (i.e., quadratic relationship), so will NUE decrease [6]; and as NUE decreases, environmental implications increase. Thus, expressing NUE as an average over-simplifies the relationship between N rate and NUE. Expressing NUE as an average could even mislead by not disclosing how low NUE is with N rates near optimal yield levels. The reality is each additional unit of N fertilizer induces a smaller and smaller yield response and at some point, environmental risks may exceed economic benefits. Here we expand on the previously introduced idea of iNUE [6, 7]. Incremental NUE means to express NUE over small increments of N fertilizer (e.g., per kg ha^-1^). We propose that expressing NUE of harvested grain N in small increments allows for exploring the dynamics of crop NUE. Further, we propose iNUE could be used as a metric in N management evaluations and development of new recommendations for producers. This investigation used extensive maize grain response to N rate datasets to: 1) quantify iNUE over typical fertilization rates; 2) relate iNUE to agronomic and environmental factors; and 3) use iNUE to propose ideas for how maize fertilizer management could be modified to balance economic and environmental goals.

## Materials and methods

Three corn N response datasets were used for this analysis (Table 1). Combined, this dataset provided 189 site-years of corn N response curves from varying soil spatial scales (i.e., regional responses throughout North America to multiple within field responses), temporal climates, corn hybrids, and management practices.

**Table 1:** Summary information of the three datasets used in this analysis.

Study	Summary	Years	Site-years used in analysis	Crop Rotation	Time of N application	N Source	Citation
North America (NA)	Study from Mexico to Canada	2006 to 2010	28	All but a few were corn following soybean	V4 to V10	Urea based fertilizer	[15]
Nebraska (NE)	Continuous plot research with fixed rotation	1991 to 2004	75	39 site-years were corn following corn, and the rest were corn following soybean	V9	Urea-ammonium nitrate	[16]
Missouri (MO)	Study measuring within field variability	2004 to 2007	86	All but a few were corn following soybean	V7 to V11	Urea-ammonium nitrate + Agrotain	[17]

### Determining the economic optimally N rates

For each site-year of data, the grain yield in response to fertilizer rates was calculated using a quadratic-plateau modeling method. For site-years to be included in the analysis, the quadratic-plateau model had to meet several criteria. First, the *F*-test needed to be significant (α = 0.10). Second, the quadratic-plateau model had to have r^2^ values ≥ 0.30. Third, the joint point for the plateau had to occur at N rates lower than the highest N rate applied for that site-year. For sites that meet these criteria, the EONR values were calculated using the first derivative of the quadratic-plateau model:

$$EONR=\frac{ratio-b}{2a}$$ (1)

Where a [in (kg grain * ha) (kg N^2^)^-1^] and b [in (kg grain) (kg N)^-1^] were the quadratic and linear coefficients, respectively. The ratio was fixed at 5.6 and derived using the cost of N fertilizer ($0.88 kg N^-1^; or $0.40 lb N^-1^) divided by the price of corn ($0.158 kg grain^-1^; or $4.00 bu grain^-1^).

### Calculating incremental N use efficiency and related metrics

For each site-year, two N use efficiency (NUE) values were calculated: an incremental NUE (iNUE) and an average NUE. Both values were based on the ratio of grain-N removal to applied fertilizer (kg grain N) (kg N)^-1^. The grain-N removed was calculated as the crop yield (kg ha^-1^) multiplied by a fixed grain N content of 0.0115 (kg grain N) (kg grain)^-1^. We used the grain N content coefficient that was reported by [18] which were the average values measured across the US North Central region. In addition, we tested the sensitivity of the average NUE to a range of grain N content coefficients from 0.010 to 0.013 (kg grain N) (kg grain)^-1^ (S1 Fig).

The iNUE, or the rate of increase in NUE for each unit of fertilizer applied, was calculated using the first derivative of the quadratic-plateau model as shown in Eq 2 for each 1 kg N ha^-1^ increment from 0 kg N ha^-1^ to EONR.


$$Incremental\;NUE=\frac{\left(b\;\times \;0.0115\right)+\left(2\;\times \;a\;\times \;0.0115\;\times \;Nrate\right)}{1\;kg\;N\;{ha}^{-1}}$$ (2)

The average NUE values were calculated as the increase in grain-N over corn that received no fertilizer per unit of fertilizer applied:

$$NUE=\frac{{{Grain\;N}_{Nrate}\;-\;Grain\;N}_{0}}{Nrate}$$ (3)

where GrainN_Nrate and_ GrainN_N0_ were the predicted grain N (kg grain N ha^-1^) for each of the N rates between 0 and EONR.

#### Calculating forgone profit relative to EONR

A partial profit was calculated using the total profit ($0.158 kg grain^-1^) minus the cost of N ($0.88 kg N^-1^) for each N rate from 0 kg N ha^-1^ to EONR. For each N rate, forgone profit or ‘unrealized profit’ (in $ ha^-1^) was calculated by dividing the partial profit at each N rate by the partial profit at EONR.

#### Predicting incremental NUE with soil and weather factors

For predicting the rate of decrease for iNUE with added N fertilizer, the slope term of each site-year was classified as either “High” or “Low” based on the overall all-site median value of 0.608. For all models (discussed below) the slopes were predicted as a function of weather and soil information. Weather and soil information were collected from publicly available datasets using Daymet [1 km gridded weather; via the ‘daymetr’ r package] and NRCS SSURGO [via the ‘soilDB’ r package]. For each unique site-year, the daily minimum and maximum daily temperature (°C) and daily precipitation (mm) were downloaded from April 15^th^ to September 15^th^. Using the daily temperature and precipitation values, additional features were derived as described by [18] that included the total precipitation, a Shannon Diversity Index [SDI; a measurement of evenness], an abundant and well distributed rainfall (AWDR), corn heat units (CHU), and growing degree days (GDD; base of 10°C). These features were calculated for three additional crop growing season time periods: the establishment phase (April 15—June 1), growth phase (June 2—July 15), and grain filling phase (July 16—September 15). Soil variables for each site-year included texture (percent sand and clay) to a depth of 30 cm, texture classification, drainage class (6 different classification ranging from poor to well drained), and taxonomic order. In addition, site management variables were also included such as irrigation (“yes” or “no”), tillage (“yes” or “no”), and crop rotation (“continuous corn”, or “corn-soybean”).

We trained the models on 60% of the data (n = 113)—a training dataset randomly selected from all the observations—and validated the models on the remaining 40% (n = 75). One site-year (from Mexico) was excluded because the soil information was inaccessible. Training the models included tunning the hyperparameters (parameters used to optimize learning) using a 10-fold cross validation repeated five times. Where the dataset was randomly divided into 10 equal sized folds. Using nine of the ten folds, multiple models were trained using each possible hyperparameter and then tested on the tenth fold. This was repeated until every fold was used as a testing fold. The process was further repeated five more times—each time the data was randomly divided into ten new folds—until there were 50 testing folds. Optimal hyperparameter values were selected based on the highest average accuracy across all 50 testing folds. The accuracy of the models was determined based on the number of correct predictions divided by the total number of observations. For the random forest model, we used the ‘randomforest’ and ‘caret’ packages with the R statistical software to tune the number of variables considered at each split. The most important variables were determined using the mean decrease in the Gini index.

To provide an example of how these variables could predict the slope terms, a recursive partitioning decision tree was fit in the same manner as the random forest (i.e., same training dataset, random seed, and variables) using the “rpart” and “mlr” packages. The hyperparameters optimized for this model included the complexity parameter (determines how much improvement to accuracy is required to keep a split), the minimum number of observations required to attempt additional splits, the minimum number of observations allowed in each terminal node, and the maximum depth of the tree.

The accuracy of the trained and tunned models was tested using the validation dataset. The accuracy was again calculated as the number of correct predictions divided by the total number of observations.

## Results

**Fig 1:**
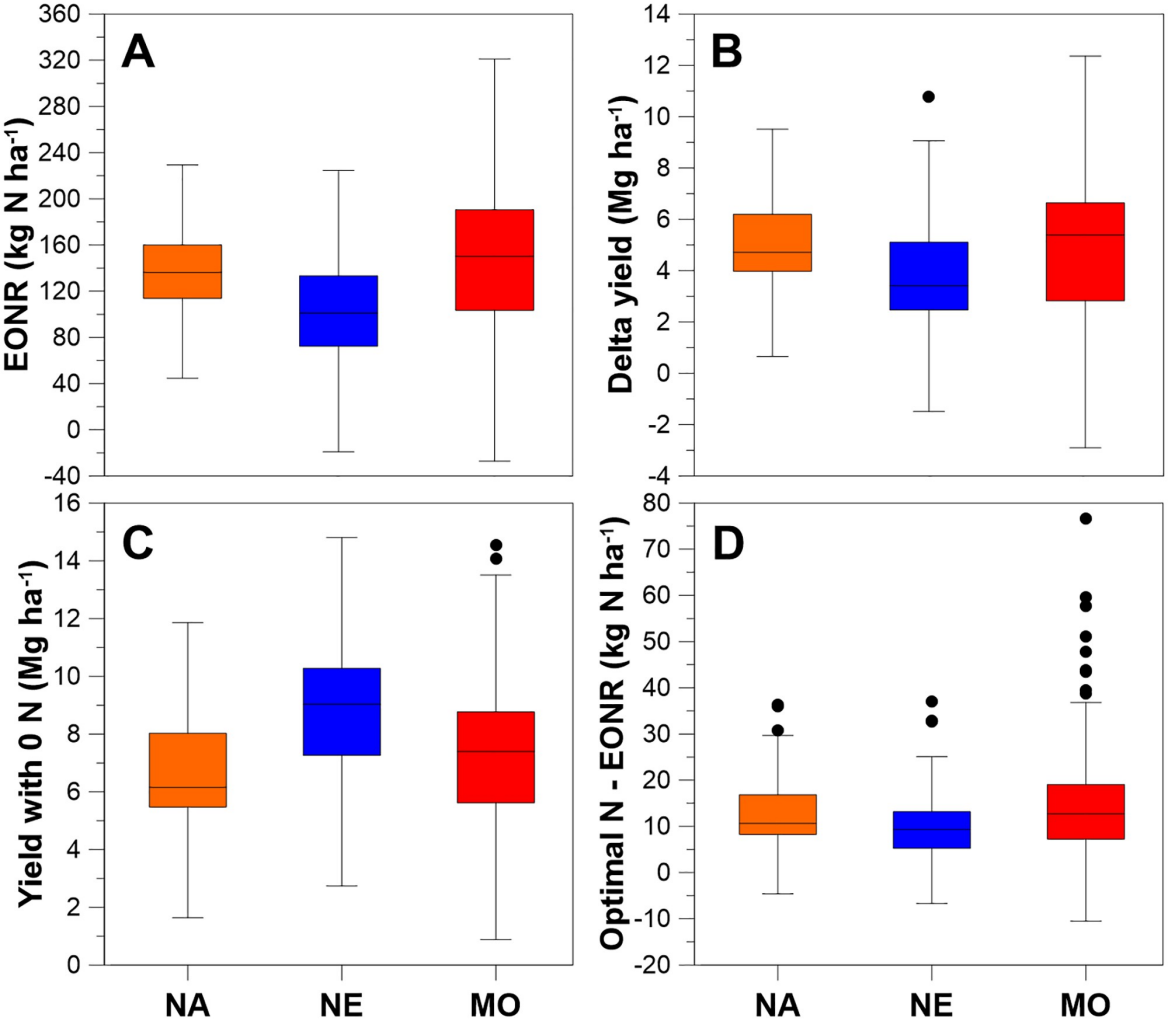
Maize productivity is highly dependent on nitrogen fertilization. Contrasted across the three datasets: (A) EONR, (B) yield increase or delta yield from EONR fertilization, (C) yield without nitrogen fertilization, and (D) the difference between optimal nitrogen rate and EONR. Box limits indicate the first and third quartile, whiskers indicate 1.5 times the interquartile range, points indicate outliers, and box line is the median.

Economic optimal N rate occurs when marginal profit equals zero. At this fertilization rate the increased return from grain sales equals the increased fertilizer cost. Any level of N application short of EONR will experience a forgone profit, defined as the decrease in profit from applying less N [19]. On average, EONR was 132, 104, and 147 kg ha^-1^ for the [15] NA, [16] NE [17], MO datasets, respectively (weighted mean = 127 kg ha^-1^; Fig 1A). Within and between datasets, the range in EONR highlights the challenge of predicting N fertilizer need with maize production. The spans of EONR were 110, 146, and 202 kg ha^-1^ for NA, NE, and MO, respectively (weighted mean = 166 kg ha^-1^). Compared to non-fertilized maize, the grain yield increase at EONR averaged 86%, 59%, and 91% for the NA, NE, and MO datasets, respectively (Fig 1B and 1C), illustrating how valuable N fertilization is in modern maize production. The difference between optimal N rate and EONR (Fig 1D) averaged 14, 10, and 16 kg ha^-1^ for NA, NE, and MO datasets, respectively (weighted mean = 14 kg ha^-1^). EONR values of these datasets were comparable to some sites reported for maize [20, 21], but less than other sites [20, 22].

**Fig 2:**
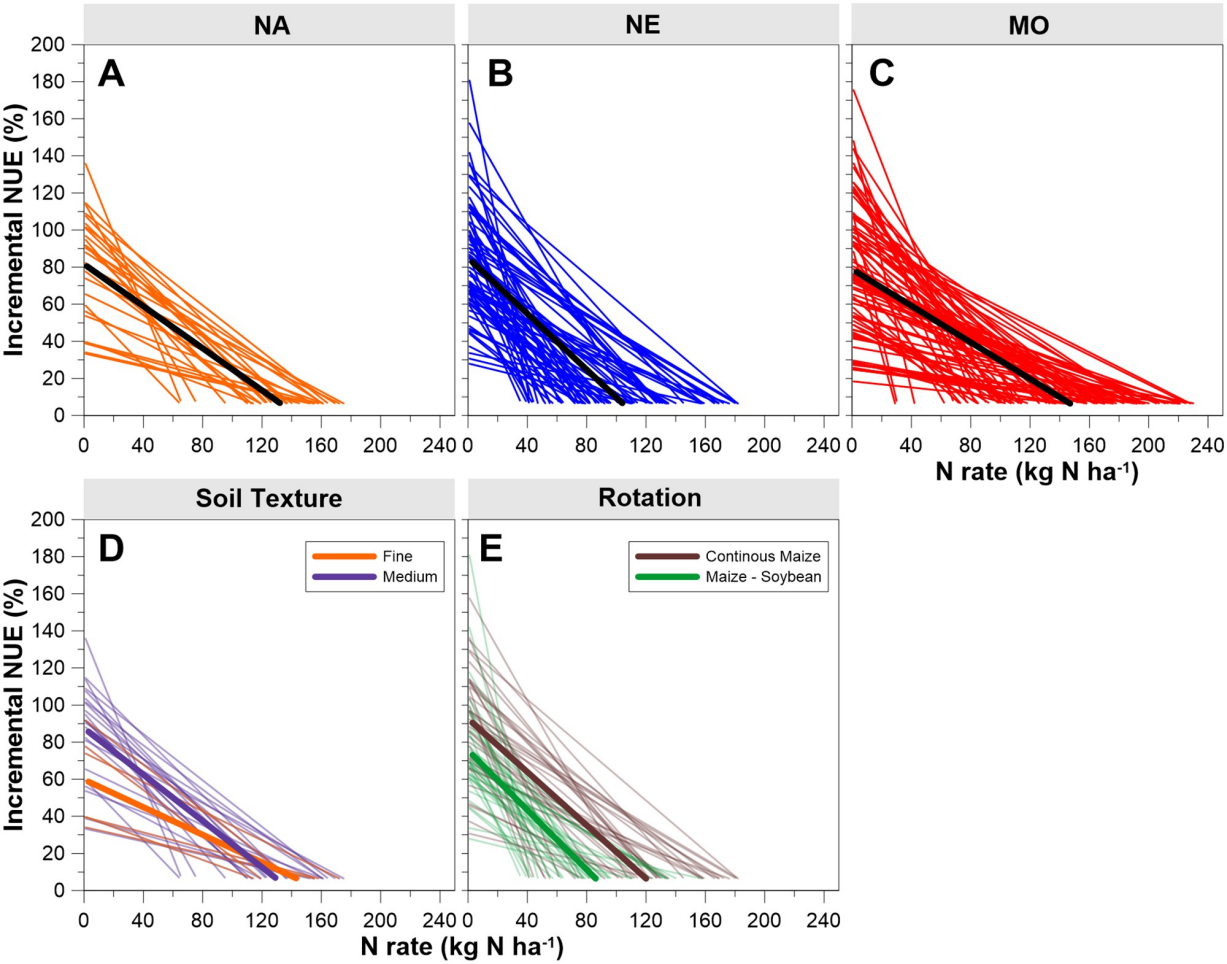
For maize, iNUE relative to increasing nitrogen fertilizer rate is not static. (A-C) iNUE shown for three datasets. For the NA dataset, iNUE at low nitrogen rates was higher for medium-textured vs fine-textured soils (D). For the NE dataset, iNUE was higher for continuous maize compared to maize rotated with soybean (E). With each graph, a line represents one site-year. The black and solid colored lines indicate the best-fit linear lines.

Utilizing EONR values obtained from quadratic-plateau relationships for these three datasets, iNUE was calculated and graphed as a function of N rates (Fig 2A–2C) with each site-year shown as an individual line on the graphs. Features amongst these site-year lines are quite variable, yet they have both agronomic and environmental meaning. When the amount of N applied was near 0 (e.g., ~1 kg ha^-1^ since undefined at 0) iNUE for that site-year was highest. Average iNUE from the first unit of fertilization was 82%, 85%, and 79% for the NA, NE, and MO datasets, respectively (weighted mean = 82%). These values show that even when a minor amount of N fertilizer is applied, typically an amount well-under N needed for optimal maize growth, an average of ~18% will be unrecovered in the harvested grain for that growing season. Many site-years (29, 25, and 28% of sites for NA, NE, and MO, respectively) had initial iNUE exceeding 100%. These represent conditions where initial amounts of fertilization stimulated N availability from organic sources through mineralization, a well-known priming-effect documented by others [12, 23]. In contrast, some site-years had initial iNUE values below 40%. We presume initial low iNUE are the result of three potential factors: 1) excessive rainfall during the growing season, causing significant fertilizer N loss (e.g., denitrification, leaching); 2) high amounts of residual mineral N prior to fertilization; or 3) other yield-limiting factors creating crop stress (e.g., soil-water deficiency, pest, other nutrient deficiencies other than N) and suppressing yield response to N. In the case of water-deficiency stress, this was presumed minor since NE sites were under irrigation and sites from the other datasets that experienced noticeable water stress were discarded entirely [15, 17].

Relative to increasing N rate, each line (Fig 2A–2C) ends at each site-year’s respective EONR value. Further, each line ends at an iNUE value of 6.4%, a value set by the fixed corn and fertilizer prices used for determining EONR. Adjusting typical prices has a nominal impact on this ending iNUE value. For example, adjusting the price of N fertilizer 40% less than that used here would alter the ending iNUE to 3.8%. If N prices were 40% more, then the ending iNUE would be 9.0%. Since in the recent decade fertilizer N prices often track maize grain prices [24], the ratio of maize to grain prices remains relatively constant, and for our analysis, the 6.4% value would be typical for US maize production. This demonstrates how low iNUE is when full fertilization is at EONR and at common maize and fertilizer prices. Stated differently, for those last few kg of N fertilizer applied to reach EONR in maize, the equivalent of less than 10% of added N would be removed in the grain. Conversely, over 90% was either lost to the environment during the growing season, remains in the soil as inorganic N subject to environmental loss without intervention (e.g., cover crops), or has been captured within maize stover and roots or soil organic matter pools.

Initial iNUE and EONR define the line that represents the decrease in iNUE with fertilization. For each of the three datasets, using the average initial iNUE and average EONR provides an average rate of decrease in iNUE for each additional kg of N applied of 0.57, 0.75, and 0.49% for NA, NE, and MO, respectively (weighted mean = 0.61%). Soil, weather, and management practices that produce crop N responses with higher initial iNUE (e.g., >80%) and relative lower EONR (e.g., < 100 kg ha^-1^) are scenarios where a greater percentage of fertilizer N is captured into the harvested grain; therefore, such practices also produce greater overall fertilizer NUE (discussed more later). In contrast, low initial iNUE tended to have higher EONR, and therefore lower rates of decrease in iNUE. These scenarios cause overall low NUE and knowing causal factors for such would be helpful in targeting management practices and public policy.

Importantly, the interpretation for these varying rates of decrease in iNUE is that NUE is not a static concept, though typically presented as such [9–11, 13, 25]. Declining iNUE with fertilization stems from increased transformation and/or loss opportunities with higher concentrations of reactive soil N. Maize studies have documented that as N fertilizer application rate exceeds optimal N rate, environmental losses increase [3, 26].

### iNUE by soil texture and crop rotation

Maize response to N fertilizer and environmental loss depends on soil properties (including soil N supply and water storage), management, and weather factors [3, 12, 27, 28]. How these factors influence iNUE is illustrated with two examples using these same datasets. Within the NA dataset, soils as previously classified [15] were found to have an average initial iNUE of 88% for medium-textured soils (n = 22) and 60% for fine-textured soils (n = 6) (Fig 2D). Only sites with medium-textured soils resulted in initial iNUE greater than 95%. Incremental NUE decrease averaged 0.63% for medium-textured soils and 0.37% for fine-textured soils. Average EONR was 116 and 140 kg N ha^-1^ for medium- and fine-textured soils, respectively. This demonstrates that with fine-textured soils, a greater equivalent portion of fertilization near EONR is unrecovered in the grain. In a previous analysis with this same dataset [15], greater amounts of N fertilizer were required to reach optimal maize grain yield for fine-textured soils compared to medium-textured soils. As explained there and elsewhere [28, 29], fine-textured soils are those with higher clay content and consequently, more predisposed to denitrification losses and/or lower mineralization. Furthermore, drainage with these soils is often poor, causing an anaerobic condition which leads to stunted early-season root development that limits late-season water uptake [30]. We attribute these as reasons iNUE differed by soil texture.

The second example contrasts maize response to N fertilization with two common crop rotations that include maize: continuous maize and maize rotated with soybean (*Glycine max*; Fig 2E). Using the NE dataset, continuous maize averaged 93% for initial iNUE, 0.72% for iNUE decrease with fertilization and 120 kg ha^-1^ for EONR. These same metrics for maize in rotation with soybean were 76%, 0.80%, and 86 kg ha^-1^, respectively. When soybean is included in a crop rotation with maize, multiple benefits have been found, including N fixation, net soil mineralization, improved conditions for seed germination, diversified microorganism community, disrupted disease cycles, and increased pest resistance [31]. Here, including soybean in the rotation translated into lower EONR and greater rates of decrease in iNUE (i.e., more efficient system). Additionally, when evaluating long-term impacts of N fertilization on NUE over many growing seasons, it is notable that fields with a maize-soybean rotation receive N fertilization half the time.

### iNUE decrease related to soil and weather

**Fig 3:**
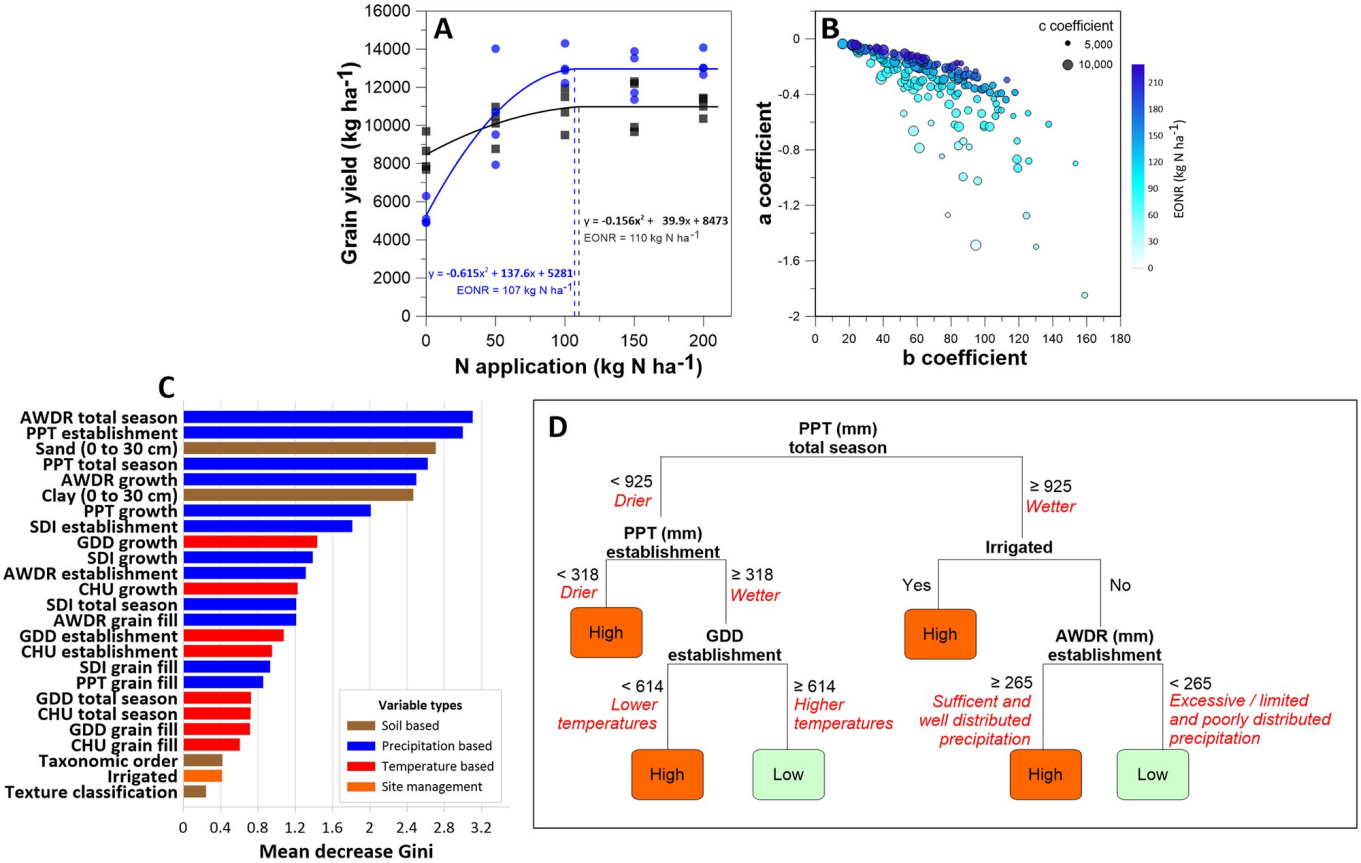
Parameters of the quadratic response model are valuable for explaining weather and soil influence on iNUE. (A) Two examples from the same site (NE dataset) of maize yield response to nitrogen fertilization (black squares = 1999, blue circles = 2000). For both, the quadratic-plateau model fits the measured yield response well, yet differently; they have similar EONR values but different quadratic-plateau model coefficients. (B) Bubble plot of all three datasets combined showing the relationships between the quadratic-plateau model terms (a, b, and c) and EONR. Low values for “a” coincide with low values for “b” (r^2^ = 0.46); and low values for “a” are related to high EONR (r^2^ = 0.50). (C) The response model terms are valuable for understanding causal relationships of weather, soil, and management to iNUE. Using a random forest model, important weather and soil variables were identified that predict the rate of iNUE decrease (i.e., slope coefficients), classed as “High” or “Low”. Rates of decrease were derived using the 1st derivative of the quadratic-plateau model and converted into grain-N units (2 x “a” quadric term x 0.0115) and classified as “High” or “Low” based on the median value of -0.608. Weather variables were calculated across different time periods: total season (April 15-September 15), emergence (April 15-June 1), growth (June 2-July 15), and grain fill (July 16-September 15). (D) An example of decision tree predicting slopes (68% accuracy on a withheld testing portion of the data) as “High” or “Low” using weather information, which included the PPT (cumulative precipitation) during establishment and for the entire season, GDD (growing degree days) during establishment, and AWDR (abundant and well distributed rainfall) during establishment.

The combined three datasets were further examined using decision tree analysis for how the decrease in iNUE was influenced by soil and weather factors (Fig 3). Two quadratic response models provide examples to contrast how maize N responses differ at the same location over two years, even under irrigation (Fig 3A). Characteristics of a response curve vary spatially and temporally based on the soil properties, weather conditions, management practices, and crop genotypes. Response model terms (Fig 3B) are not only related to each other, but also can be used to understand causal relationships of multiple weather and soil factors to iNUE decrease (Fig 3C). Precipitation factors dominated in importance (blue), but soil (brown) and temperature (red) factors also helped explain iNUE decrease. For this analysis, management information availability was minimal, and therefore contributed little. The example decision tree (Fig 3D) demonstrates the critical nature water amount and distribution have on iNUE decrease. Conditions with too much rainfall and/or uneven rainfall produced a “Low” decrease in iNUE. Producers realize this from experience, and it is uncertain weather that largely drives adding extra N fertilizer as insurance [32, 33]. Since the forecasted weather trends for the US Midwest are for greater annual precipitation, more of the annual total in the spring, and more variable summer rainfall [34], improved weather forecasting offers one of the best options for improving N management and subsequently improving NUE. Doing so will require producers embrace more adaptive practices.

### Profit relative to iNUE

**Fig 4:**
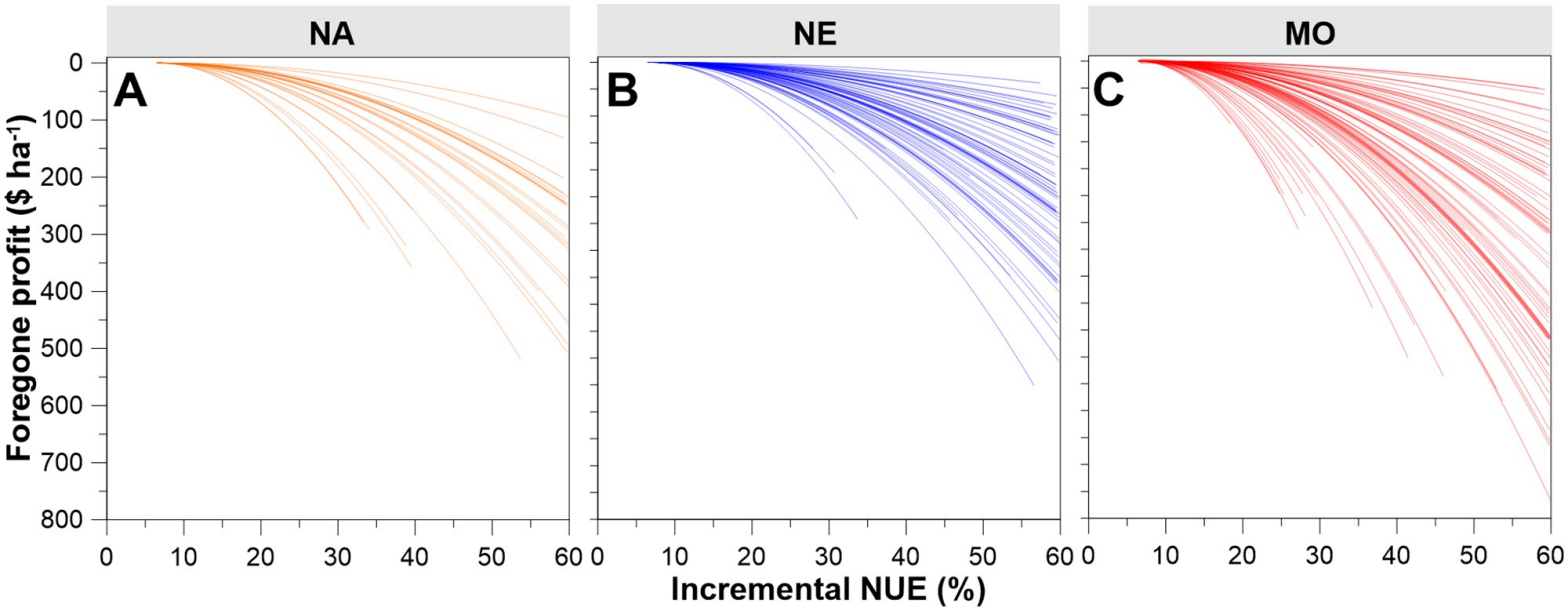
Foregone profit (relative to EONR) is minimal when iNUE is low. (A-C) Foregone profit for each of the three datasets, calculated as the profit difference at iNUE and economically optimal nitrogen rate (EONR). iNUE was limited to < 60%. Each line represents one site-year.

EONR is characterized by low iNUE. There is no forgone profit from N rate decisions when fertilization is at EONR and iNUE is 6.4%. As shown in Fig 4, this relationship presents forgone profit relative to iNUE and is least at low iNUE. Forgone profit increases exponentially as iNUE increases. Because of convergence of site-years when marginal profit nears zero, forgone profit is more alike amongst site-years at low iNUE, and more dissimilar as iNUE increases. Using this relationship, the influence of forgone profit with sub-EONR fertilization can be further explored.

**Fig 5:**
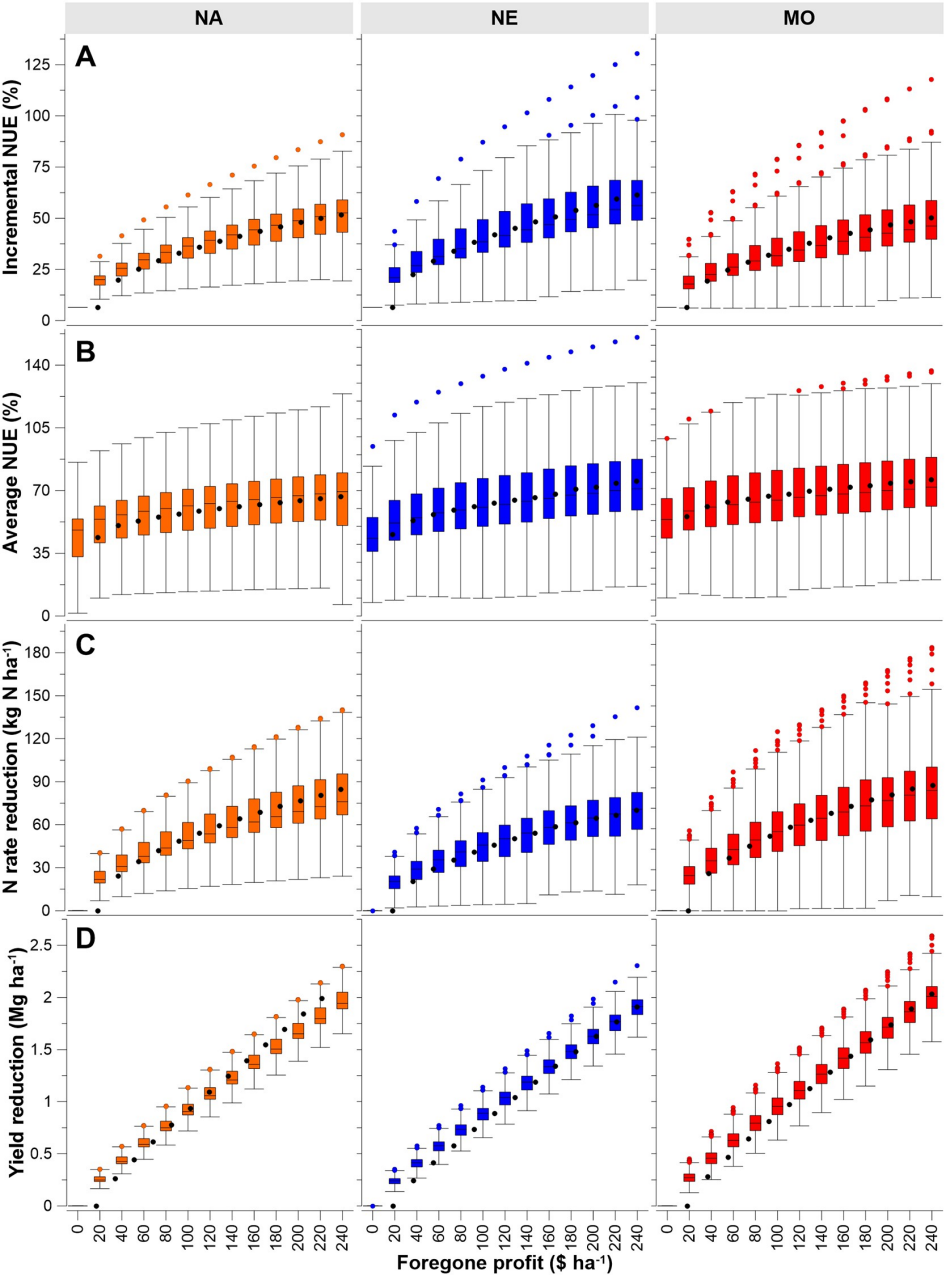
Realize large improvements in average NUE by removing low iNUE. (A) Incremental NUE for the three datasets shown corresponding to foregone profit. (B) Average NUE [(grain Nx–grain N0)/x; x = N rate)] shown relative to foregone profit. (C) Nitrogen saved (EONR- N rate) shown relative to foregone profit. (D) Yield reduction (or loss) with sub-EONR fertilization shown relative to foregone profit. With each graph, box limits are the first and third quartile, whiskers indicate 1.5 times the interquartile range, circles indicate outliers, the box line is the median, and the black circle is the mean.

Distributions of site-year iNUE were summarized relative to forgone profit at $20 ha^-1^ step values from EONR to sub-EONR rates (Fig 5A). With each additional $20 ha^-1^ forgone profit step, iNUE values and their ranges increase notably. The most significant change in iNUE is with the first $20 ha^-1^ ($8.10 ac^-1^) step of forgone profit, with iNUE increasing by 14.3 percentage points, from 6.4% to 20.7% (weighted mean). The interpretation of this is that minor sub-EONR fertilization eliminates the lowest iNUE values for the smallest forgone profit; further deviation from EONR eliminates less iNUE for the same amount of forgone profit. On average, forgone profit of $20 ha^-1^ from sub-EONR fertilization effectively excludes all iNUE values from 6.4% up to 20.7%. When unrealized profit is set to $120 ha^-1^ ($48.5 acre^-1^), average iNUE values were 39, 45, and 38% for NA, NE, and MO datasets, respectively, and a weighted mean of = 41%. Thus, at this profit reduction, iNUE values < 41% are effectively removed. Because of the quadratic relationship of maize response to N, changes in iNUE decrease as forgone profit increases. For a profit reduction of $240 ha^-1^ ($97.1 acre^-1^) from sub-EONR fertilization, average iNUE values from 6.4% up to 55% (weighted mean) are eliminated (52, 61, and 50% for NA, NE, and MO datasets, respectively).

How does removing low iNUE values change the average NUE [i.e., average NUE = [(grain N_x_ − grain N_0_)/x; x = N rate)]? Using the same foregone profit steps, distributions of average NUE are illustrated (Fig 5B). At EONR, average NUE was 44, 45, and 43% for NA, NE, and MO datasets, respectively, with an overall mean average of 44%. These values are comparable to other reported average NUE values [9, 10, 12, 13]. Average NUE increased to 50, 53, and 49% (weighted mean = 51%) with only $20 ha^-1^ foregone profit, illustrating the impact of low iNUE values near EONR on overall average NUE. Average NUE as the weighted mean across datasets increased to 61% with sub-EONR fertilization at a $120 ha^-1^ foregone profit, and to 69% with a $240 ha^-1^ foregone profit.

**Fig 6:**
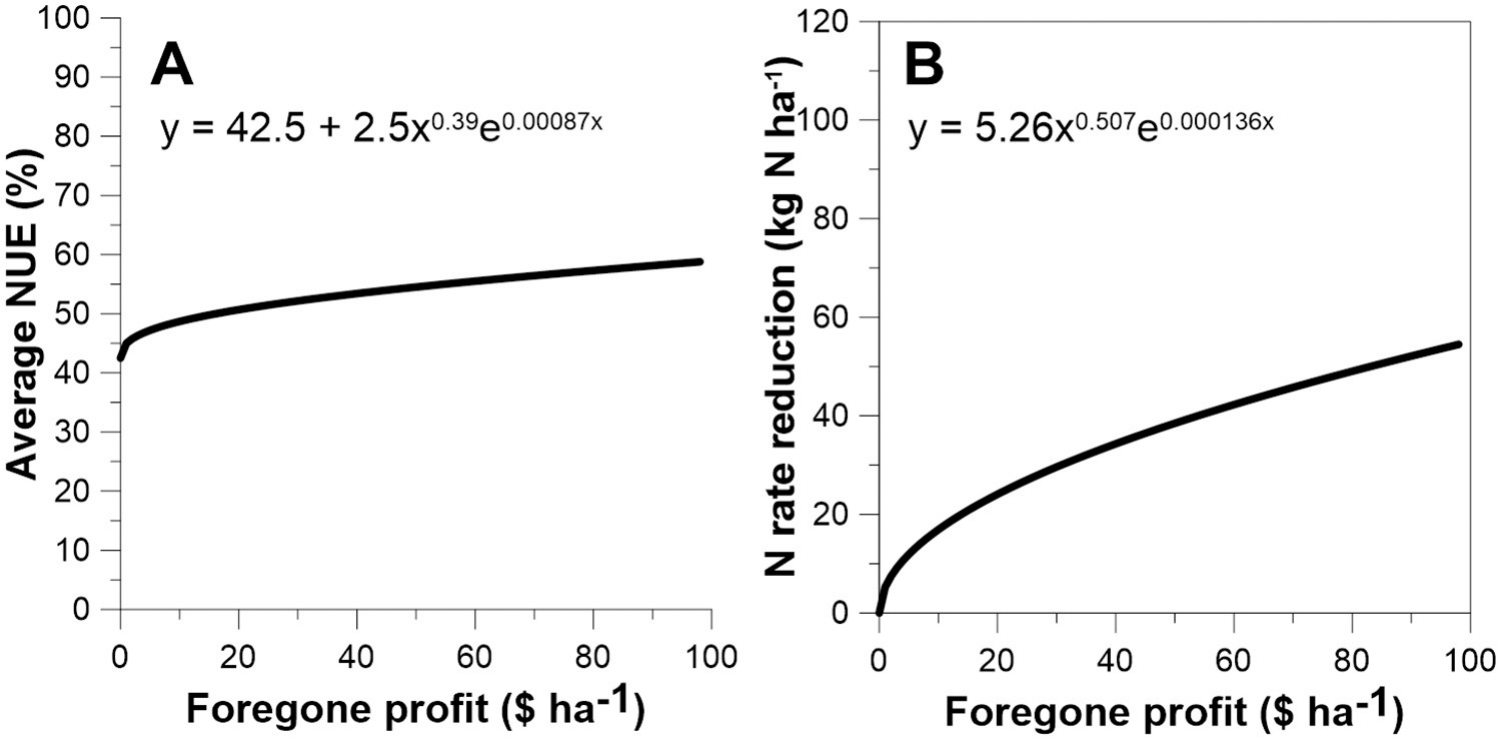
With modest sub-EONR fertilization, average NUE improvements of ~10% can be realized. (A) Averaged over the three studies, this equation shows the average NUE shown relative to foregone profit when N fertilization is less than EONR. (B) Model showing the average N saved with sub-EONR fertilization relative to foregone profit.

Nitrogen fertilizer saved at the same foregone profit steps is shown in Fig 5C. For the first step, reducing N below EONR by 24 kg N ha^-1^ (Fig 5C) bolstered average NUE by 7 percentage points (Fig 5B). Initial sub-EONR fertilization improved incremental and average NUE the most, and with minor foregone profit. Because foregone profit is minor, yield reduction too is minor (Fig 5D). A summary across datasets is presented in Fig 6.

## Discussion

Can iNUE be used to improve N management? First, we suggest that examining NUE incrementally helps expose the need for exploring more sustainable N fertilizer practices. Nitrogen management includes many interdependent choices (e.g., crop rotation, genetics, and fertilizer timing, source, placement, stabilizers), but when considering environmental implications, the choice of fertilizer amount is typically most impactful since the standard of supporting decision tools is to apply at EONR [12]. The iNUE analysis provided shows that fertilization at EONR amounts is problematic environmentally, but also creates a unique perspective for influencing change. Potential outcomes from giving attention to iNUE will be discussed.

First, if low iNUE exists at EONR, then the problem is exacerbated when rates exceed EONR, as is the case when insurance N applications are made [32, 33]. Such application rates push iNUE near or at 0%. If adding extra insurance N is not an appropriate answer, how then do maize producers deal with year-to-year uncertainty associated with weather and water availability? Decision tools that capture interactions of in-season weather and soil (e.g., process-based crop growth modeling) can help manage this uncertainty, but refinement is needed with the crop growth models [12], soil representations [15], and weather forecasting [35]. Further, reliance on in-season applications will require improvements in infrastructure for fertilizer supply, delivery, and standing-crop applications.

Is EONR the right target for N fertilizer rate management? While held as the standard for decades [12], it ignores low iNUE, as illustrated. Yet, current N rate decision tools built upon economics as the standard could easily be modified to embrace environmental objectives, such as using the average NUE to foregone profit relationship shown in Fig 6A. Maize producers are generally risk adverse, unlikely to embrace foregone profit from sub-EONR applications without compensatory incentives. Since all society would be beneficiaries of improved crop NUE, we encourage support of the development and enhancement of N decision support tools that allow consideration of incentive payments offered to producers to match anticipated foregone profit from sub-EONR applications. Ideal decision support tools should incorporate the following concepts. One, reasonable and accurate estimates of EONR that are specific to soils and multiple growing-season weather conditions need to be known, similar to the Maximum Return to N (MRTN) database that exists for many parts of the US Midwest Cornbelt region [12]. Two, producers would be required to adopt management practices known to improve NUE, such as applying most N within season and using sources of N along with stabilizers that minimize N loss [12]. Third, the approach would need to guard against inflation of anticipated EONR values, so producers are not drawing incentive payments and still fertilizing to optimal yield levels. Localized EONR values each year may be needed to establish this, perhaps through a certified third party. Utilizing precision agriculture technologies can greatly facilitate this need [36]. Using modern combine harvesters with calibrated yield-monitoring systems would help establish sub-EONR fertilization by field or farm. For example, a field fertilized at presumed sub-EONR could also include several EONR or EONR+ monitoring strips. To make this approach somewhat universal, the targeted sub-EONR rate would likely be set to some percentage of EONR. For example, data for this analysis shows 85% of EONR would represent ~20 kg N ha^-1^ reduction from EONR (Fig 6). Yield mapped data of these monitoring strips to adjacent maize would provide documentation to trigger incentive payment. If yield was comparable between the strips and adjacent maize, then sub-EONR application was not achieved, and no incentive payment would be made. If the rest of the field yielded less, then sub-EONR applications could be concluded and an incentive payment, to some reasonable amount, made to match the foregone profit and extra effort for implementation. To test and refine this decision support approach, a pilot research program that applies these tools should start in soil regions historically shown to have a high propensity for environmental N loss.

Distributions represented by site-years as shown in Fig 5A–5C demonstrate that embracing a single universal relationship (e.g., Fig 6) would be less than ideal. The dominant factor driving variation in soil N and crop N need are soil properties (e.g., texture, drainage, landscape water redistribution) interacting with weather [15, 37–39]. This soil by weather interaction causes within-field yield-stability differences, and understanding these interactions help characterize within-field variable NUE [4]. Additional soil and management factors such as rotation, tillage, genetics, and fertilizer source, timing, and placement are also known to influence maize response to N rate [12], and therefore also impact iNUE (Fig 3C and 3D). To ignore these factors over-simplifies the challenge associated with crop N management. These factors should be considered within EONR estimations to add precision. Some factors are already included in some degree with EONR databases (e.g., MRTN by local soil regions [12]), but many are not. To the extent possible, soil/weather/management factors should be quantified for how they influence EONR, and in turn their quantitative influence on iNUE.

Crop production practices in place for decades rarely change quickly, but cultural norms can be altered through formal and informal educational activities. We propose a need for educational outreach programs that focus on environmental implications through the metric of iNUE. Specifically, with crop N management, lack of producer awareness of the environmental implications is a major barrier for altering practices [40, 41]. Since iNUE is a product of an economic production function (i.e., EONR), producers will quickly connect to the implications captured with iNUE, perhaps more so than other environmental N loss metrics such as leaching and denitrification. Also, decision aids developed for producers for making fertilizer recommendations should incorporate anticipated iNUE.

## Conclusions

Economics drive decisions of how much N fertilizer to apply when it is so crucial to yield, fertilizer is relatively inexpensive, and weather is uncertain. Thus, switching from a single economic-based objective to dual objectives where environmental consequences are also included typically requires intervention. Governmental intervention approaches have been tried, including limiting amounts use per cropped area or by incentivizing, such as with a fertilizer tax [19]. Yet, effectiveness of these approaches varies widely because of differences in producer risk aversion [42]. Utilizing iNUE to address environmental goals would be unique to previous utilized interventions since it starts with the economic crop response function to develop the environmental response function. As shown here, influences of weather, soil, and management factors can easily be characterized within iNUE, giving site-specific sensitivity that N management requires. Such promotes efficiencies to be gained by utilizing newer technologies through precision agriculture [36, 39, 43]. Most importantly, iNUE allows for simultaneous consideration of N management practices to achieve a balance between economic and environmental goals.

## Supporting information

S1 Table Global production of major grains exceeded 2613 million metric tons in 2020 (from Statista 2019/20; available at https://www.statista.com/statistics/263977/world-grain-production-by-type). Tabulated N removed in grain based on representative crude protein content from the Food and Agriculture Organization (FAO).
(DOCX)

S2 Table Six common approaches to expressing NUE. These largely have evolved based on the kind of information that was readily available and how the potential for the calculations enhanced interpretation of data. The yield component in these calculations is a common metric; however, users need to express the water content basis of the grain. For example, maize grain marketed through an elevator will be adjusted to 15.0 or 15.5% moisture content. However, the grain N concentration is commonly measured using an oven-dried sample, so calculation of grain N content needs to be adjusted to the same water content.
(DOCX)

S3 Table Example maize yield, biomass, and various NUE calculations at five fertilizer N rates. Data from 2003 Nebraska dataset (see Table 1). Interpreting NUE can be misleading because lack of any N fertilizer is still likely to produce some grain provided water is adequate to support plant growth. Yet some NUE calculations do not consider grain yield when no N fertilizer is applied. Perhaps the most intuitive NUE calculation is to compare N removed in grain with fertilizer N applications. Data inputs (yield and amount of N fertilizer applied) are easy to acquire and grain N concentration can be estimated with good reliability. Each NUE calculation has a decreasing value as the N rate increases. The exception is termed “producer efficiency” because the values are used by producers to help assess the efficiency of N management practices.
(DOCX)

S1 Fig Grain nitrogen content effects calculations for average NUE. For the analysis on the three datasets for this paper, maize nitrogen content of 11.5 g kg^-1^ was used as published (*1*). Relative to this value, using grain nitrogen content numbers less or more than this have been used by others, and would decrease or increase average NUE, respectively. Average NUE for 10 g nitrogen (kg grain)^-1^ and NA, NE, and MO datasets would be 38.1, 39.5, and 36.9%, respectively. Average NUE for 11.5 g nitrogen (kg grain)^-1^ and NA, NE, and MO datasets would be 43.8, 45.4, and 42.5%, respectively. Average NUE for 12 g nitrogen (kg grain)^-1^ and NA, NE, and MO datasets would be 45.7, 47.4, and 44.3%, respectively. Average NUE for 13 g nitrogen (kg grain)^-1^ and NA, NE, and MO datasets would be 49.6, 51.3, and 48.0%, respectively.
(TIF)

S1 Dataset Raw and processed data from three corn N response datasets used for this analysis. (XLSX)
