# Supplementary material for: A new perspective when examining maize fertilizer nitrogen use efficiency, incrementally
Source: PLoS One. 2022 May 11;17(5):e0267215. doi: 10.1371/journal.pone.0267215 (PMC9094541; doi:10.1371/journal.pone.0267215)
Supplement: S1 Table — Tabulated N removed in grain based on representative crude protein content from the Food and Agriculture Organization (FAO). (DOCX) [file pone.0267215.s001.docx]

| Grain Type | Production | Crude Protein | Grain N |
| --- | --- | --- | --- |
|  | (mil. metric ton) | (%) | (mil. metric ton) |
| Maize | 1116.34 | 9.3 | 16.61 |
| Wheat | 764.49 | 12.7 | 15.53 |
| Rice | 495.78 | 8.4 | 6.66 |
| Barley | 156.41 | 11.6 | 2.90 |
| Sorghum | 57.97 | 11.0 | 1.02 |
| Oats | 22.83 | 11.8 | 0.43 |
| TOTAL | 2613.82 |  | 43.15 |
|  |  |  |  |
