# Supplementary material for: A new perspective when examining maize fertilizer nitrogen use efficiency, incrementally
Source: PLoS One. 2022 May 11;17(5):e0267215. doi: 10.1371/journal.pone.0267215 (PMC9094541; doi:10.1371/journal.pone.0267215)
Supplement: S2 Table — These largely have evolved based on the kind of information that was readily available and how the potential for the calculations enhanced interpretation of data. The yield component in these calculations is a common metric; however, users need to express the water content basis of the grain. For example, maize grain marketed through an elevator will be adjusted to 15.0 or 15.5% moisture content. However, the grain N concentration is commonly measured using an oven-dried sample, so calculation of grain N content needs to be adjusted to the same water content. (DOCX) [file pone.0267215.s002.docx]

| Agronomic Efficiency (AE) | (kg grain - ck grain/kg N fertilizer) | |  |  |
| --- | --- | --- | --- | --- |
| Partial Factor Productivity (PFP) | (kg grain/kg N fertilizer) |  |  |  |
| Producer Efficiency | (lb N fertilizer/bu grain) |  |  |  |
| Partial N Balance (PNB) | (kg grain N/kg N fertilizer) |  |  |  |
| Uptake Efficiency (UE) | (kg N uptake-ck N uptake/kg N fertilizer) | |  |  |
| Recovery Efficiency (RE) | (kg grain N - ck grain N/kg N fertilizer) | |  |  |
