# Supplementary material for: A new perspective when examining maize fertilizer nitrogen use efficiency, incrementally
Source: PLoS One. 2022 May 11;17(5):e0267215. doi: 10.1371/journal.pone.0267215 (PMC9094541; doi:10.1371/journal.pone.0267215)
Supplement: S3 Table — Data from 2003 Nebraska dataset (see Table 1). Interpreting NUE can be misleading because lack of any N fertilizer is still likely to produce some grain provided water is adequate to support plant growth. Yet some NUE calculations do not consider grain yield when no N fertilizer is applied. Perhaps the most intuitive NUE calculation is to compare N removed in grain with fertilizer N applications. Data inputs (yield and amount of N fertilizer applied) are easy to acquire and grain N concentration can be estimated with good reliability. Each NUE calculation has a decreasing value as the N rate increases. The exception is termed “producer efficiency” because the values are used by producers to help assess the efficiency of N management practices. (DOCX) [file pone.0267215.s003.docx]

| Fertilizer N Rate (kg/ha) | 0 | 50 | 100 | 150 | 200 |
| --- | --- | --- | --- | --- | --- |
|  |  |  |  |  |  |
| Yield (Mg/ha)(dry) | 3.489 | 6.91 | 10.25 | 11.282 | 11.933 |
| Yield (Mg/ha)(@ 15.5%)) | 4.129 | 8.178 | 12.130 | 13.351 | 14.122 |
| Grain N (g/kg) | 12 | 12 | 12 | 12 | 12 |
| Grain N removed (kg/Mg) | 49.548 | 98.130 | 145.562 | 160.218 | 169.463 |
| Total biomass (Mg/ha) | 11.813 | 17.413 | 22.677 | 23.898 | 25.141 |
| Total residue N uptake (kg/ha) | 58.268 | 73.521 | 86.989 | 88.312 | 92.456 |
|  |  |  |  |  |  |
| Agronomic Efficiency |  | 81.0 | 80.0 | 61.5 | 50.0 |
| (kg grain - ck grain/kg N) |  |  |  |  |  |
| Partial Factor Productivity |  | 163.6 | 121.3 | 89.0 | 70.6 |
| (kg grain/kg N) |  |  |  |  |  |
| Producer Efficiency |  | 0.34 | 0.46 | 0.63 | 0.79 |
| (lb N/bu) |  |  |  |  |  |
| Partial N Balance |  | 1.96 | 1.46 | 1.07 | 0.85 |
| (kg grain N/kg N) |  |  |  |  |  |
| Uptake Efficiency *(total)* |  | 1.28 | 1.25 | 0.94 | 0.77 |
| (kg N uptake-ck N uptake/kg N) |  |  |  |  |  |
| Recovery Efficiency *(grain)* |  | 0.97 | 0.96 | 0.74 | 0.60 |
| (kg grain N - ck grain N/kg N) |  |  |  |  |  |
