# Supplementary figures and images for: A new perspective when examining maize fertilizer nitrogen use efficiency, incrementally
Source: PLoS One. 2022 May 11;17(5):e0267215. doi: 10.1371/journal.pone.0267215 (PMC9094541; doi:10.1371/journal.pone.0267215)

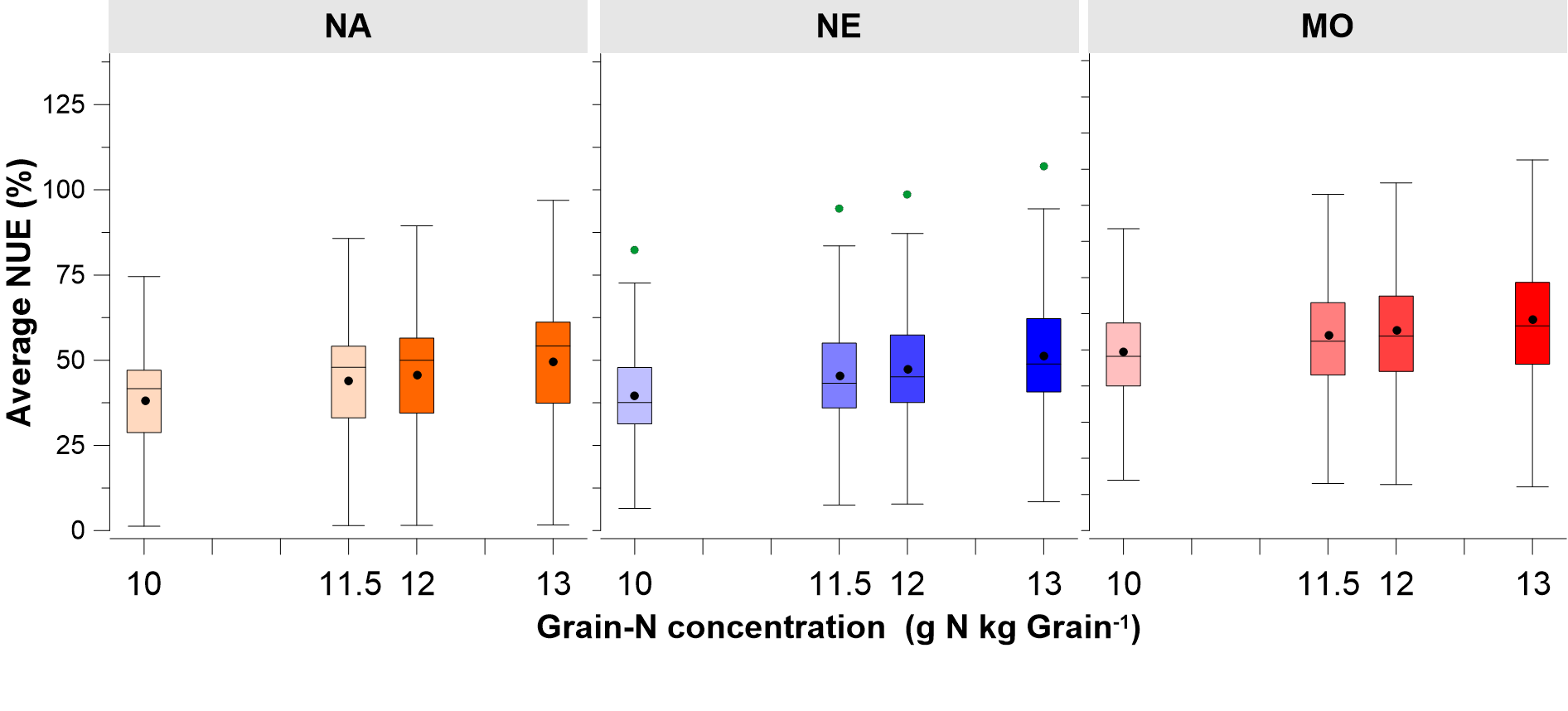

Supplement: S1 Fig — For the analysis on the three datasets for this paper, maize nitrogen content of 11.5 g kg-1 was used as published (1). Relative to this value, using grain nitrogen content numbers less or more than this have been used by others, and would decrease or increase average NUE, respectively. Average NUE for 10 g nitrogen (kg grain)-1 and NA, NE, and MO datasets would be 38.1, 39.5, and 36.9%, respectively. Average NUE for 11.5 g nitrogen (kg grain)-1 and NA, NE, and MO datasets would be 43.8, 45.4, and 42.5%, respectively. Average NUE for 12 g nitrogen (kg grain)-1 and NA, NE, and MO datasets would be 45.7, 47.4, and 44.3%, respectively. Average NUE for 13 g nitrogen (kg grain)-1 and NA, NE, and MO datasets would be 49.6, 51.3, and 48.0%, respectively. (TIF) [file pone.0267215.s004.tif]
